# Emeritus Professor F. H. Edgeworth

**Published:** 1943

**Authors:** 


### Obituary

### EMERITUS PROFESSOR F. H. EDGEWORTH, M.A., M.D. (Cantab.), D.Sc. (Lond.).

By the passing onwards of Francis Henry Edgeworth the medical profession has lost one of its most learned members, and the scientific world has been deprived of an eminent and indefatigable research worker. The son of Dr. T. F. Edgeworth, and beginning his education at Clifton College, Edgeworth went on to Caius College, Cambridge, with a scholarship, and secured a first in the Natural Science Tripos, graduating in 1887, and becoming Shuttleworth Scholar of Caius. He then entered the Bristol Medical School, taking his M.B., B.C. (Cantab.), thence proceeding to Paris, Gottingen, London, and Dublin for further medical training. He became D.Sc. (Lond.) in 1903, and M.D. (Cantab.) in 1908, was honorary pathologist to Bristol Royal Infirmary from 1890 to 1895, and was appointed Assistant Physician in 1893, being elected Physician in 1906, which post he held until 1924, when he became an Honorary Consulting Physician. In 1905 he became Professor of Medicine in University College, Bristol, and later occupied the Chair of Medicine in the University, being appointed Emeritus Professor on his retirement. During the Great War he served in the R.A.M.C. from 1914 to 1918, being attached to the 2nd Southern General Hospital, and retiring with the rank of Major.

Edgeworth published many valuable monographs in the realms of science and medicine, but his *opus magnum* was a volume published in 1935 by the Cambridge University Press and entitled *The Cranial Muscles of Vertebrates.* This publication involved a colossal amount of work in the preparation and microscopic examination of sections from various types of embryo, and also the execution of a very large number of drawings made from these sections. A considerable amount of time was spent by him on the construction of large models, made to render him assistance in arriving at his conclusions. In the course of this work he realized that Mind played an important part in directing embryonic growth. In some unknown way the growing organism appears to forsee future needs and sets to work with such material as it has to develop the necessary structures along the lines followed by its ancestors. Experimental embryology shows that the growing organism, from the zygote upwards, is a whole, and not merely an aggregate of parts. Similarly animals are psychological units characterized by memory and purpose by striving after ends in view. The variations in the development of homologous structures, and in the structures by which the same function is carried out show that this psychological factor is of great importance from the very first. This immaterial, non-spatial, teleological factor, the Mind, can initiate and inhibit physico-chemical processes. Life, development of individual organisms, and evolution are primarily due to this power. As to the origin of Mind he was an opponent of epiphenomenalism, and a disciple of Plato.

In his work as a physician Edgeworth recognized that medicine was primarily a clinical science and relied very largely on his own observations made at the bedside, seeking the assistance of laboratory workers to help in or confirm his own diagnosis, rather than make one for him. He adopted this attitude with the knowledge that a clinical examination systematically conducted is as much scientific as the determination of blood-sugar or the identification of bacteria in sputa. All his patients were treated with the same degree of skill and with the greatest kindness, no distinction being made between Dives and Lazarus. It is not easy to say in what branch of medicine Edgeworth was most expert, but it was probably in the difficult art of differential diagnosis, especially in relation to diseases of the central nervous system.

In 1896 Edgeworth married Miss Ethel Maude Usher, a lady of considerable intellectual attainments, who was in complete sympathy with all his scientific aspirations, and encouraged and helped him in his work in every possible way. She predeceased him in 1931, and one of the chief objects of his life disappeared with her demise. The dedicatory words to her memory which appear in his book speak for themselves regarding the part she played in his life. "In memoriam uxoris meae quae mihi, ut in ceteris rebus, ita in hoc opere et socia erat et adiutrix." Edgeworth never ceased in his endeavour to add to the sum of human knowledge, and quite failed to recognize his own attainments. He leaves a son, a naturalized American citizen, at present serving with the forces of the country of his adoption.

O. C. M. D.

